# Premature Burial

**Published:** 1900-09

**Authors:** 


### *EDITORIAL NOTES AND COMMENTS.*

The Business office of THE JOURNAL-RECORD is 305, 306 Fitten Building.

The Editorial office is 318-319-320 The Prudential.

Address all Business communications to Dr. M. B. Hutchins, Mgr.

Make remittances payable to THE ATLANTA JOURNAL-RECORD OF MEDICINE.

On matters pertaining to the Editorial and Original Communications address Dr. Bernard Wolff, Atlanta.

Reprints of original articles will be furnished at cost price. Requests for the same should always be made *on the manuscript.*

We will present, post-paid, on request, to each contributor of an original article, twenty (20) marked copies of THE JOURNAL-RECORD containing such article.

### PREMATURE BURIAL.

AN American society for the prevention of premature burial has recently been incorporated. It contains some well-known names among its members, who are members of the Medico-Legal Society, from whose loins the new society has sprung. The intent of the society is implied by the name. It is for rescuing those who are dead, yet alive; mortal, yet immortal. Just how the rescue work is to be accomplished is not made clear, unless the French invention of a sort of post-mortem annunciator, a patent Gabriel's trump, is to be considered.

Has there ever been an authentic case of premature burial, except that of Lazarus, whose reanimation is classed among the Christine miracles?

"As one who on a lonesome road
Doth walk in fear and dread,
And having once turned round, walks on
And turns no more his head;
Because he knows a fearful fiend
Doth close behind him tread."

What more gruesome imagery could be conjured up to the timorous wayfarer than the picture of the buried-alive straining at his narrow cell in a vain attempt to escape from a double death. This fine scene is a favorite among "goblins-will-get-you" reporters and rural correspondents, but it lacks substantiation. Narrow escapes are frequently chronicled. Mark Twain tells of a good one in the course of the description of Jim Blaine's ram, and there are others.

But for the real thing, not the sham, is premature burial possible? The puncture of the abdomen to allow the escape of gas, the injection of the body with some preservative, bichloride or formula solution through one of the important vessels, the retention of the body for a reasonable length of time before interment, then the air-tight coffin and six feet of earth. Isn't this enough to kill in itself? If life remained while this familiar routine were begun it would not long survive. Under such circumstances the undertaker kills and the condition is premature death, not burial. D'Arsonval insisted that the post-mortem on the "electrocuted" was the cause of death, not the electric current. It does not matter; the result is the same. There is merely a shifting of the functions of "M. de Paris" from the man at the switch to the pathologist.

Perhaps this is treating a grave subject (pun intentional) with too much flippancy, yet it is mildly diverting to witness the incorporation of a society for the prevention of spook and hobgoblin fictions.

Why not engage, after Mrs. Jellyby, in a more practical enterprise like that of the distribution of foot-tubs among the Boers, or of eau de cologne among the Georgia niggers.

> "These shall pursue the helpless dead,
> These shall the helpless dead pursue."

